# Zero-Biased Photoelectrochemical Detection of Cardiac Biomarker Myoglobin Based on CdSeS/ZnS Quantum Dots and Barium Titanate Perovskite

**DOI:** 10.3390/molecules27154778

**Published:** 2022-07-26

**Authors:** Fernanda M. R. Lima, Alan S. de Menezes, Adeilton P. Maciel, Francisco S. M. Sinfrônio, Lauro T. Kubota, Flávio S. Damos, Rita C. S. Luz

**Affiliations:** 1 Department of Chemistry, Federal University of Maranhão, São Luís 65080-805, Brazil; fernanda.m20@hotmail.com (F.M.R.L.); ap.maciel@ufma.br (A.P.M.); 2 Department of Physics, Federal University of Maranhão, São Luís 65080-805, Brazil; almez13@yahoo.com.br; 3 Department of Electrical Engineering, Federal University of Maranhão, São Luís 65080-805, Brazil; kjvida@mac.com; 4 Institute of Chemistry, State University of Campinas, Campinas 13083-970, Brazil; kubota@unicamp.br

**Keywords:** immunosensor, photoelectrochemistry, myoglobin, barium titanate, CdSeS/ZnS quantum dots

## Abstract

Cardiovascular diseases are considered one of the leading causes of premature mortality of patients worldwide. Therefore, rapid diagnosis of these diseases is crucial to ensure the patient’s survival. During a heart attack or severe muscle damage, myoglobin is rapidly released in the body to constitute itself as a precise biomarker of acute myocardial infarction. Thus, we described the photoelectrochemical immunosensor development to detect myoglobin. It was based on fluorine-doped tin oxide modified with CdSeS/ZnSe quantum dots and barium titanate (BTO), designated as CdSeS/ZnSQDS/BTO. It was characterized by scanning electron microscopy (SEM), energy-dispersive spectroscopy (EDX), transmission electron microscopy (TEM), X-ray diffraction (XRD), electrochemical impedance spectroscopy (EIS), and amperometry. The anodic photocurrent at the potential of 0 V (vs. Ag/AgCl) and pH 7.4 was found linearly related to the myoglobin (Mb) concentration from 0.01 to 1000 ng mL^−1^. Furthermore, the immunosensor showed an average recovery rate of 95.7–110.7% for the determination of myoglobin.

## 1. Introduction

Myoglobin (Mb) is a protein formed by a polypeptide chain composed of 153 amino acids and found in muscle tissue, where its main function is the storage and transport of oxygen [1]. Due to its low molecular weight and extensive liberation into the blood plasma after the onset of an infarction event, such protein is one of the main cardiac biomarkers used for Acute Myocardial Infarction (AMI) diagnosis [2,3].

The normal Mb concentration in the body ranges from 30.00 to 90.00 ng mL^−1^ and increases dramatically with the onset of AMI, reaching ~200.00 ng mL^−1^ or even 900.00 ng mL^−1^ in the higher peak periods. Thus, a specific, highly sensitive, and early detection of Mb in the blood is essential for diagnosing AMI [2,4,5,6]. In this sense, many methods are reported in the literature for the detection of Mb, such as immunoenzymatic assay [7,8], fluorescence [9,10], surface plasmon resonance [11,12], mass spectrometry [13,14], and liquid chromatography [15,16]. However, although such methods have a higher selectivity for Mb quantification, they are very expensive and require long sample preparation and analysis. These limitations could be an obstacle in diagnosing patients with AMI since fast and reliable clinical decisions are desirable for managing the disease [2,17].

Conversely, methodologies based on electrochemical detection of biomarkers have shown to be promising due to their easy handling, good sensitivity, and chemical selectivity. These systems transform the interaction with specific analytes in electrochemical signals based on a biorecognition element that establishes selective interactions. These biomarkers can be enzymes, nucleic acids, peptides, or antibodies [18,19].

The photoelectrochemical immunosensors make possible a simple operation, portability, wide response range, low cost, and miniaturization. These characteristics attracted great attention in the biological area. Besides that, combining photoelectrochemical platforms with antigen-antibody interaction offers high sensitivity/selectivity of biological species [20,21]. The photoelectrochemical mechanism forms electron-hole pairs in the photoactive semiconductor materials after the incidence of electromagnetic radiation and, when captured by acceptors or electron donors, produces photocurrent [22]. In this sense, the choice of the semiconductor material in constructing the PEC system is essential for the efficiency of detecting biomolecules.

The perovskite oxides, such as barium titanate (BTO), present good chemical stability, environmental benignity, excellent optical properties, and ferroelectricity. The latter’s spontaneous polarization can improve electron-hole separation [23,24,25]. However, BTO has a gap band of 3.2 eV and absorbs mainly in the ultraviolet region of the spectrum [26]. Therefore, some strategies have been proposed to improve its ability to absorb visible light photons, including its combination with narrow band gap materials based on quantum dots.

Nanostructured quantum dots semiconductors (QDs) have interesting electrical, optical, and catalytic properties depending on their size and shape [27]. Materials such as CdSe, CdS, InAs, and CuInS_2_ can be used as photosensitizers due to their broad absorption spectra, allowing photoelectrochemical detection systems based on these materials to be excited by ordinary white light, making them attractive materials [28]. In addition, nanoparticles based on core-shell structure with inorganic capping, such as CdSe/ZnS, CdS/ZnS, and CdSe/CdS show greater efficiency concerning emission, efficient electron transport, and high carrier mobility values [29,30]. CdSeS/ZnS QDs nanocrystals are among the most studied inorganically capped nanoparticles due to their high luminescence, photostability, and lower toxicity. Therefore, the BTO was combined with the CdSeS/ZnS QDs to exploit the high stability of BaTiO_3_ perovskites and the capability of the CdSeS/ZnS QDs to harvest visible light.

In addition, electrochemical detection of molecules is usually performed under a potential sufficient to oxidize or reduce the species of interest; energy is spent. Generally, the oxidation of many molecules occurs above 0 volts, while the reduction occurs by biasing the electrode at potential values lower than 0 volts. Therefore, detecting a molecule at a potential of zero volts (under short-circuit conditions) is highly desirable. Furthermore, a low potential reduces the amount of energy required to oxidize or reduce the molecule of interest and minimizes species’ interference in the analyte response. In this work, vitamin C, denoted as VC, was used as an electron donor molecule to monitor the analytical signal of myoglobin, Mb. VC usually oxidizes at potentials above 400 mV. However, with the proposed photoelectrochemical platform, it was possible to exploit VC as a donor molecule at zero volts and consequently monitor the analytical signal of Mb with the zero-biased photoelectrochemical platform. Thus, this paper has constructed a new photoelectrochemical immunosensor based on CdSeS/ZnS core-shell QDs and BTO nanoparticles to detect Mb.

## 2. Results and Discussion

### 2.1. Characterization of the Materials by SEM, EDX, TEM, XRD and EIS

**Figure 1:**
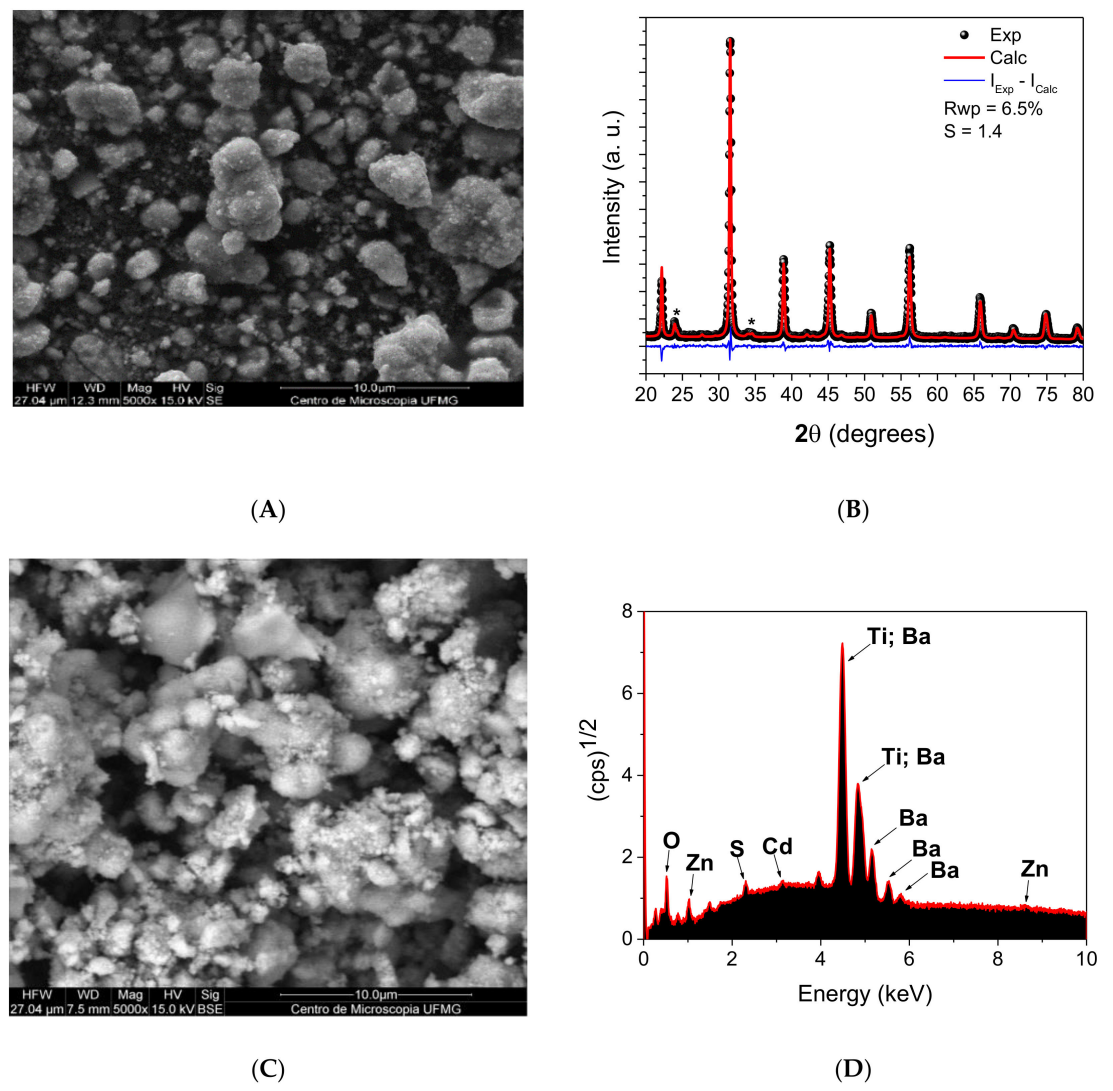
(**A**) SEM image for the BTO or BaTiO_3_ perovskite at a magnification of 5000×; (**B**) XRD patterns, with Rietveld refinement, for BTO; (**C**) SEM image for the CdSeS/ZnSQDs/BTO material at a magnification of 5000×; (**D**) EDX spectrum of the CdSeS/ZnSQDs/BTO material.

Figure 1A shows scanning electron microscopy (SEM) images for the BaTiO_3_ (BTO) at a magnification of 5000×. As can be seen, the perovskite presents particles formed by small granules. In addition, XRD patterns, with Rietveld refinement, for BTO are shown in Figure 1B. The diffractogram presents the (1 0 0), (1 1 0), (1 1 1), (2 0 0), (2 1 0), (2 1 1), (2 2 0), (3 0 0), (3 1 0) and (3 1 1) characteristic peaks of BaTiO_3_ perovskite with Cubic structure and Pm-3m Space Group (ICSD: 34900) [31]. Its structure was confirmed by Rietveld refinement and the lattice parameter a = 4.0099(1) Å, crystallite size D = 48.1(6) nm and microstrains s = 0.077(2)% were obtained. Some residue of the orthorhombic BaCO_3_ precursor used in the synthesis of BaTiO_3_ was also observed in the XRD pattern. The concentrations of the phases were also obtained from Rietveld refinement as follows: BaTiO_3_ (92.7(3)%) and BaCO_3_ (7.3(3)%). In addition, Figure 1C shows particles of a very rugous surface probably due to the presence of several clusters of quantum dots CdSeS/ZnSe deposited on the surface of larger BTO particles. In Figure 1D, almost all the peaks referring to the CdSeS/ZnSQDs/BTO material are observed except the Se element due to the difficulty of observing this element on the BTO when it is present in small quantities.

**Figure 2:**
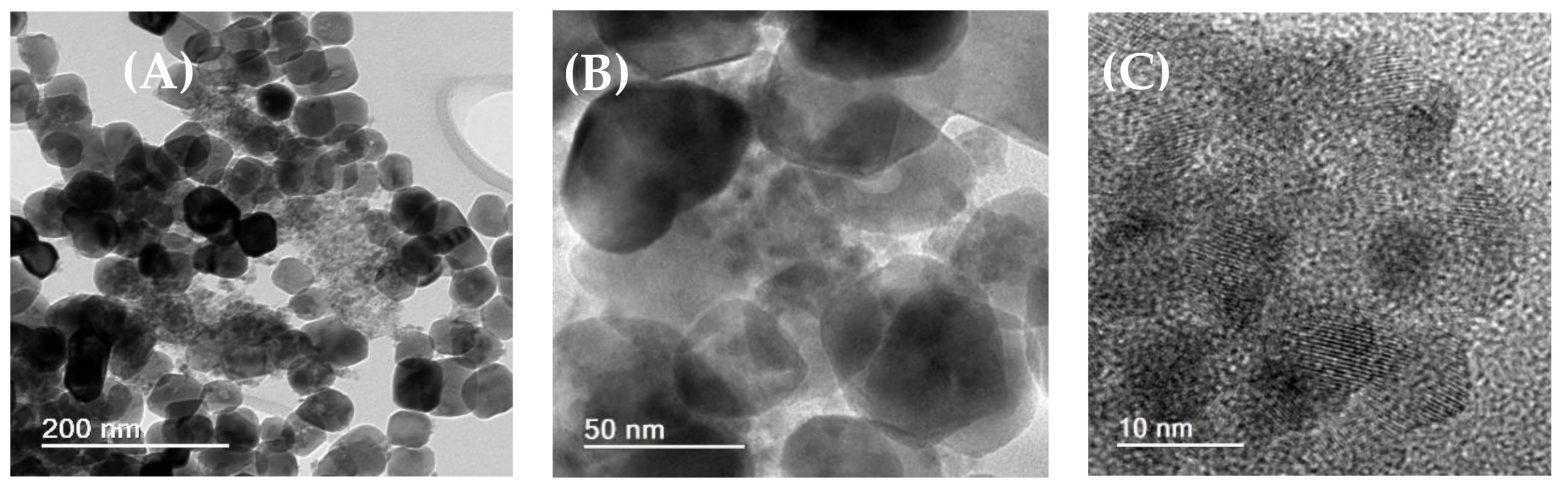
TEM images of CdSeS/ZnSQDs/BTO at three different scales: (**A**) 200 nm, (**B**) 50 nm, and (**C**) 10 nm.

Figure 2A–C show TEM images of CdSeS/ZnSQDs/BTO at scales of 200, 50 and 10 nm, respectively. The transmission electron microscopy (TEM) images confirm the presence of quantum dots and BTO in CdSeS/ZnSQDs/BTO material. Figure 2C shows a region where it is possible to observe QDs particles with a diameter lower than 10 nm.

Figure S1A–C (Supplementary Materials) show three different regions of CdSeS/ZnSQDs/BTO material. As can be seen, an interplanar spacing of 0.28 nm was observed in TEM image presented in Figure S1A indicating the presence of BTO (BaTiO_3_) [32]. On the other hand, the Figure S1B presents regions indicating the presence of particles of low size and lattice separation of 0.35 nm due to the quantum dot core [33]. In addition, both interplanar spacing of 0.28 nm and 0.35 nm were observed in the TEM image presented in Figure S1C.

### 2.2. Characterization of the Photoelectrochemical Platform and Optimization of Experimental Conditions for the CdSeS/ZnSQDs/BTO/FTO Sensor 

**Figure 3:**
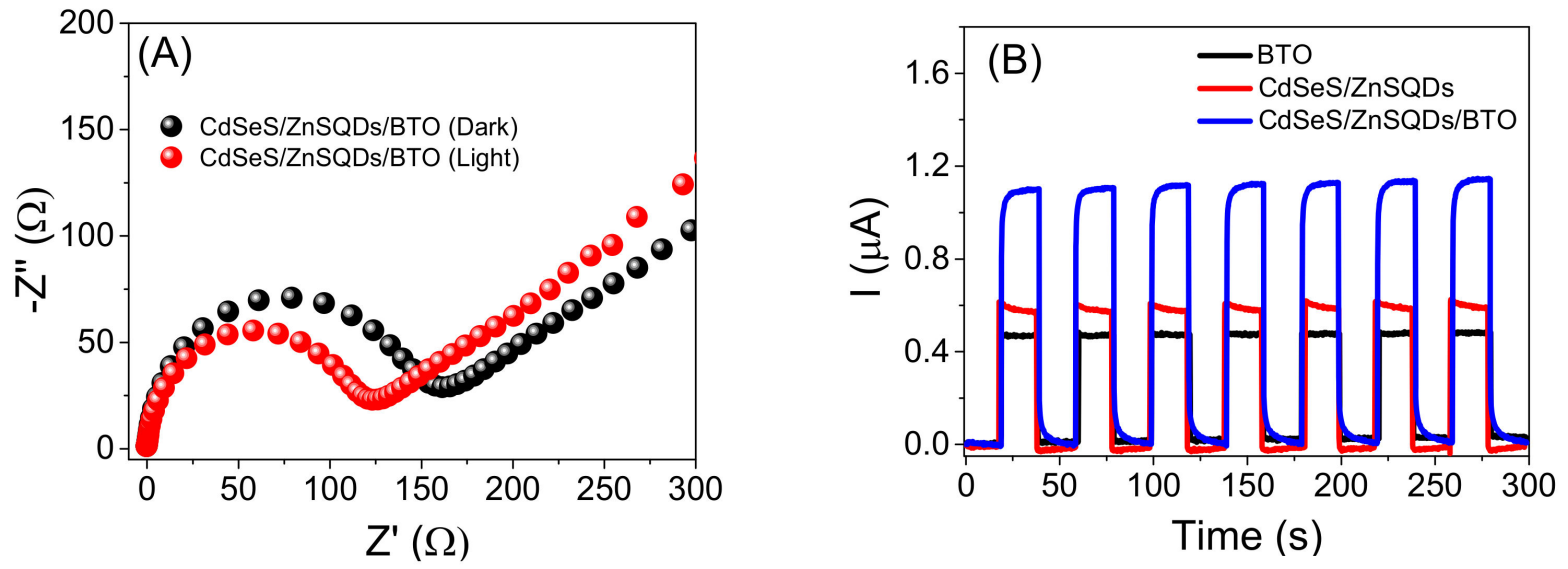
(**A**) EIS spectra of the FTO modified with CdSeS/ZnSQDs/BTO were obtained in 0.10 mol L^−1^ of potassium chloride with 0.005 mol L^−1^ of potassium ferricyanide in the presence (red balls) and absence of visible LED light (black balls); (**B**) Photocurrent obtained for the CdSeS/ZnSQDs/BTO/FTO (blue line), CdSeS/ZnSQDs/FTO (red line), and BTO/FTO (black line) in 0.10 mol L^−1^ of PBS, pH 7.4, containing 0.20 mol L^−1^ of VC, under applied potential of 0 V vs. Ag/AgCl (st. KCl).

Figure 3A shows the electrochemical impedance measurements for the FTO electrode modified with the CdSeS/ZnSQDs/BTO material. The measurements were performed in 0.10 mol L^−1^ potassium chloride solution containing 0.005 mol L^−1^ potassium ferricyanide in the absence (black balls) and presence of visible light (red balls). The used frequency range was 10^−2^–10^5^ kHz. According to it, CdSeS/ZnSQDs/BTO/FTO platform presented a charge transfer resistance of 161.40 Ω and 123.40 Ω, respectively, in the absence and presence of light. On the other hand, it is observed that light decreases the resistance to charge transfer of the electrode surface due to the presence of the CdSeS/ZnS quantum dots that facilitate the charge transfer process, increasing the photoelectrochemical sensor response (Figure 3A). The sensor response depends on the light incidence onto the electrochemical cell since the presence of light improves the number of electron-hole (e^−^/h^+^) couples. The electron-hole pairs photogenerated in the semiconductor provided a more efficient transfer of electrons to the electrode, resulting in less charge recombination. Thus, it can be concluded that the presence of light on the CdSeS/ZnSQDs/BTO generated a more effective charge separation and increased the photoelectrochemical efficiency of the complete material.

Figure 3B shows the photocurrent obtained in the presence of molecule donor (VC) for the BTO/FTO, CdSeS/ZnSQDs/FTO, and CdSeS/ZnSQDs/BTO/FTO platforms. The reached photocurrents for the CdSeS/ZnSQDs/BTO/FTO platform were about 2.43 and 1.84 times greater than those obtained for the electrode modified only with the barium titanate or with quantum dot, respectively. This enhanced photocurrent for the complete material may be assigned to better separation of the photogenerated charges and lower charge recombination for the donor molecule.

**Figure 4:**
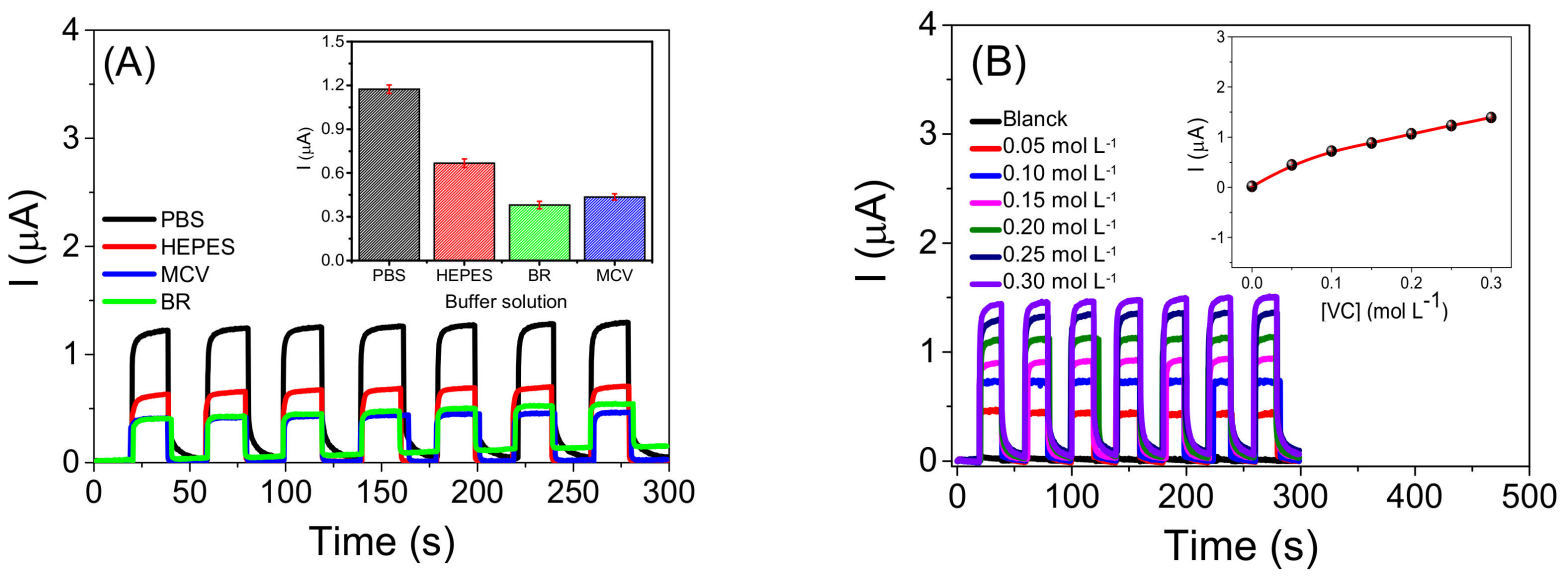

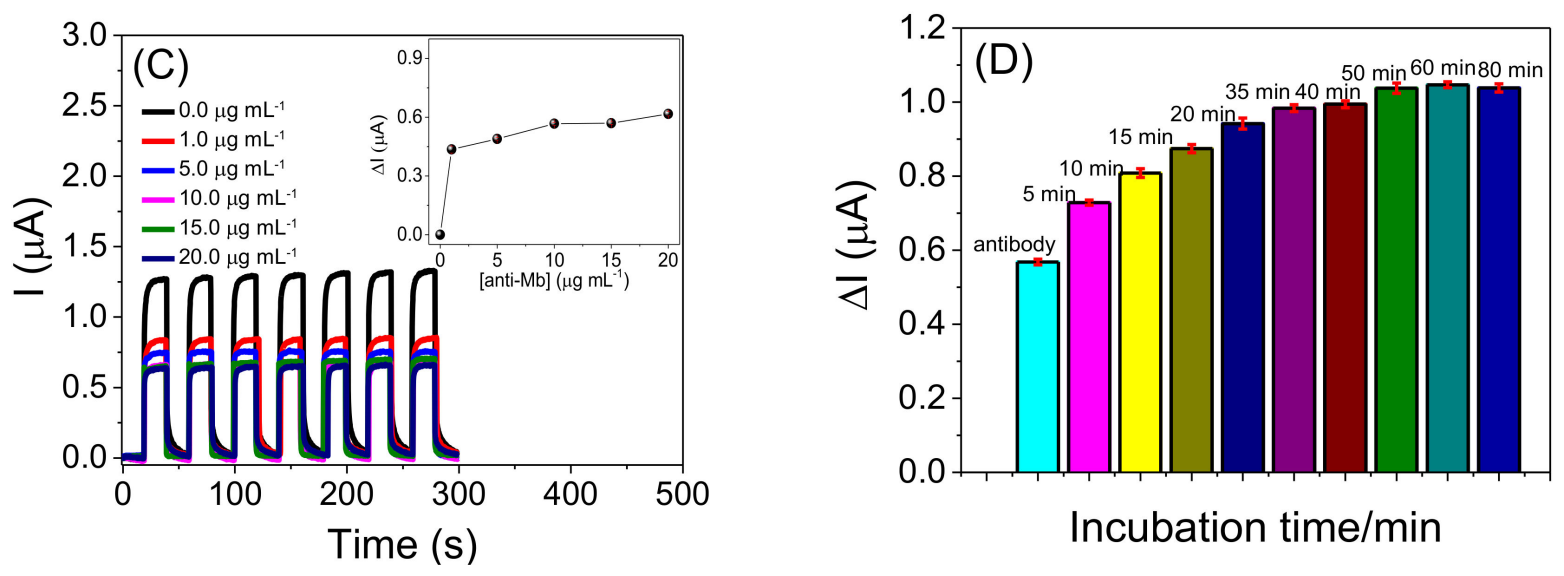
(**A**) Amperograms for the FTO modified with CdSeS/ZnSQDs/BTO in phosphate buffer solution (PBS); HEPES; Macllvain (MCV), and Britton-Robinson (BR), containing 0.20 mol L^−1^ of VC. Insertion of Figure 4A: Plot of current vs. buffer solution; (**B**) Amperograms for the CdSeS/ZnSQDs/BTO/FTO platform containing different concentrations of VC. Insertion of Figure 4B: Plot of current vs. VC concentration; (**C**) Amperograms obtained for the anti-Mb/CdSeS/ZnSQDs/BTO/FTO immunosensor at employing different concentrations of anti-Mb. Insertion of Figure 4C Plot of current vs. concentration of anti-Mb; (**D**) Plot of the anti-Mb/CdSeS/ZnSQDs/BTO/FTO immunosensor response at different times of incubation with Mb. Experiments performed 0.10 mol L^−1^ PBS, pH 7.4, containing 0.25 mol L^−1^ of VC under an applied potential of 0 V.

Thus, in order to improve the sensitivity of the CdSeS/ZnSQDs/BTO/FTO platform, the effect of the concentration of VC, the type of buffer solution, the concentration of Mb antibodies, and the immunoreaction time between anti-Mb and Mb. As shown in Figure 4A, we evaluated the photocurrent of the sensor to four support electrolytes containing 0.20 mol L^−1^ of VC. In this study, the phosphate buffer solution, PBS, HEPES solution, Britton-Robinson solution, BR, and McIlvaine solution, MCV, were prepared at 0.10 mol L^−1^ (pH 7.4). The highest current values were reached in PBS, which may be associated with the high mobility of phosphate ions in the solution due to its small size. Thus, charge transport will be more efficient between the electrode/solution interface, increasing the photocurrent response.

The influence of the VC concentration on the photoelectrochemical platform photocurrent was also evaluated (Figure 4B). Thus, the photocurrent for the CdSeS/ZnSQDs/BTO/FTO electrode was monitored in a range of 0.0–0.30 mol L^−1^ of VC in 0.10 mol L^−1^ of PBS (pH 7.4). In the inset of Figure 4B, there was a more remarkable growth in VC concentration of 0.05–0.10 mol L^−1^ and a smaller increase from 0.10–0.30 mol L^−1^ (insertion Figure 4B). However, the growth in the photocurrent value was smaller for the photoelectrochemical platform at VC concentrations of 0.25 mol L^−1^ (1.230 ± 0.009 µA) and 0.30 mol L^−1^ (1.390 ± 0.010 µA); in this sense, the concentration of 0.25 mol L^−1^ was fixed for further studies, since it presented a lower standard deviation for the measurements. 

In order to evaluate the analytical parameters that interfere in the photocurrent of the CdSeS/ZnSQDs/BTO/FTO, the platform was modified with different concentrations of anti-Mb. In addition, after the evaluation of the antibody concentration, the incubation time of the anti-Mb/CdSeS/ZnSQDs/FTO immunosensor with the Mb was also investigated. 

Figure 4C shows a photocurrent decrease in the presence of VC for the CdSeS/ZnSQDs/BTO/FTO platform after immobilizing different concentrations of anti-Mb (1–20 µg mL^−1^) in 0.10 mol L^−1^ PBS, pH 7.4. As can be seen, there was a significant decrease in the photocurrent for concentrations from 1 to 10 µg mL^−1^. However, from the concentration of 10 µg mL^−1^ there was certain stability in decreasing photocurrent in the system up to 20 µg mL^−1^. The inset of Figure 4C shows the photocurrent variation (ΔI) versus the anti-Mb concentration. ΔI represents the difference between the photocurrent obtained before and after the incubation of the immunosensor in an anti-Mb solution. However, the efficiency of the immunosensor in the presence of the donor molecule is highly affected by very high concentrations of antibody Mb. Thus, the concentration of 10 µg mL^−1^ was chosen for further studies.

Finally, the study of the interaction time between anti-Mb/CdSeS/ZnSQDs/BTO/FTO with Mb was carried out by incubating the sensor in a solution of 100 ng mL^−1^ Mb at different times of incubation: 5 to 80 min (Figure 4D). ΔI represents the difference between the photocurrent obtained before and after the incubation of the immunosensor in an Mb solution. An increase in the photocurrent is observed with the incubation time from 5 min up to 40 min. However, after 40 min, there was no significant increase in photocurrent. Thus, this time was fixed for further studies.

### 2.3. Analytical Performance of the Photoelectrochemical Immunosensor

**Figure 5:**
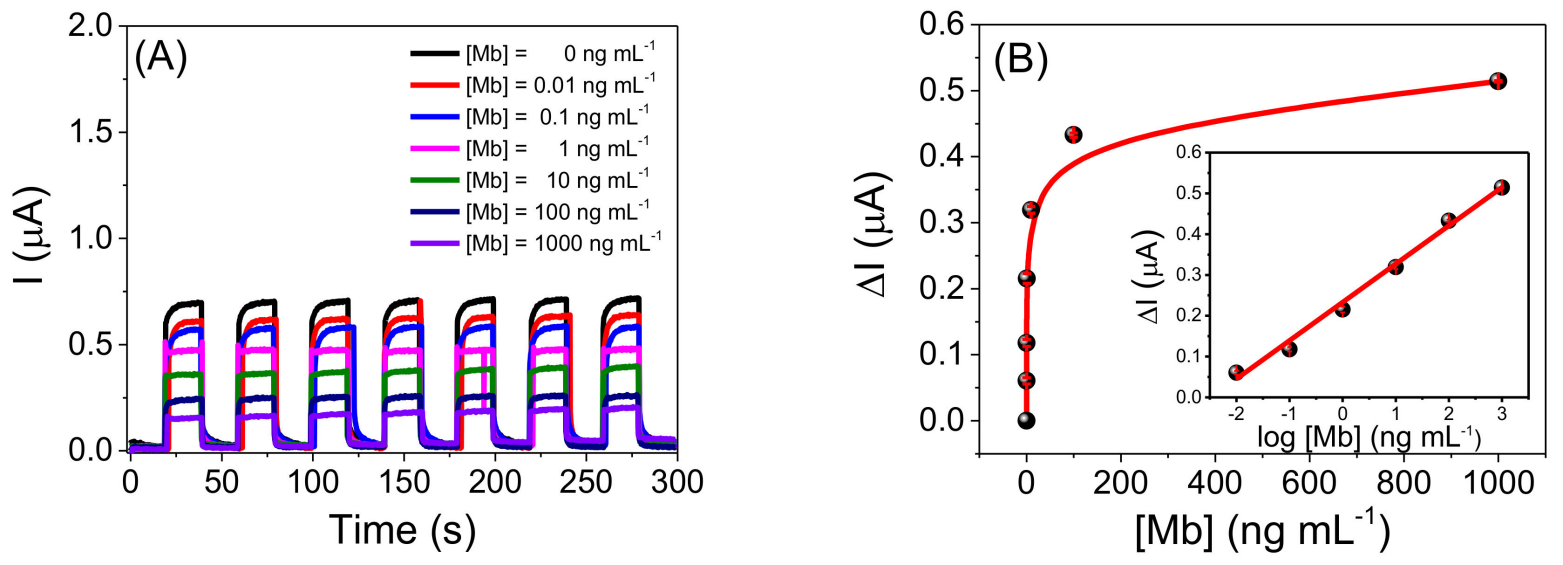
(**A**) Amperograms for the anti-Mb/CdSeS/ZnSQDs/BTO/FTO immunosensor photoelectrochemical before incubation, [Mb] = 0 ng mL^−1^, and after incubation of different [Mb] (0.01–1000 ng mL^−1^); (**B**) Plot of the variation photocurrent for the photoelectrochemical immunosensor vs. [Mb] obtained from Figure 5A. Inset of Figure 5B: Plot of the log [Mb] vs. photocurrent response obtained for the anti-Mb/CdSeS/ZnSQDs/BTO/FTO photoelectrochemical immunosensor. Experiments performed in 0.10 mol L^−1^ PBS, pH 7.4 containing 0.25 mol L^−1^ of VC applied potential of applied potential of 0 V and incubation time of 0.67 h.

The anti-Mb/CdSeS/ZnSQDs/BTO/FTO photoelectrochemical immunosensor was used to detect Mb at different concentrations (Figure 5A). In the presence of VC, a decrease in the photocurrent was probably caused by the reaction between Mb and anti-Mb on the immunosensor surface. Figure 5B shows that the photocurrent variation (ΔI) is proportional to the increase in the Mb concentrations. Thus, it was possible to build a plot of the ΔI against the logarithm of the Mb concentration from 0.01 to 1000 ng mL^−1^ (inset of Figure 4B) expressed by the following equation: ΔI (µA) = 0.233 + 0.094 log [Mb] (ng mL^−1^) with a correlation coefficient of 0.996 (*n* = 6), showing good linearity. The proposed immunosensor can experimentally detect 0.01 ng mL^−1^ (10 pg mL^−1^) of Mb. The analytical parameters obtained with the anti-Mb/CdSeS/ZnSQDs/BTO/FTO were equated with other platforms to detect Mb reported in the literature. Table S1 [3,5,11,34,35,36,37,38,39,40,41,42,43,44,45,46,47,48,49,50,51] shows the analytical characteristics of immunosensors for Mb and a comparison of materials with the platform proposed. This table shows several matrices used for the detection of Mb. As can be seen in Table S1, the proposed methods showed very satisfactory results. Some researchers find a good incubation time for Mb of up to 30 min. However, despite having chosen a time of 40 min for the myoglobin incubation using the PEC platform based on CdSeS/ZnSQDs/BTO, it was possible to determine a better LD and concentration range than all the works presented in Table S1. It is important to note that many PEC platforms developed for other molecules generally employ a high-power Xenon lamp (e.g., 500 W) and a much higher-cost photoelectrochemical system than the system used in this work. In this sense, we used a photoelectrochemical system composed of a commercial 30 W visible LED lamp and a homemade rigid box.

**Scheme 1:**
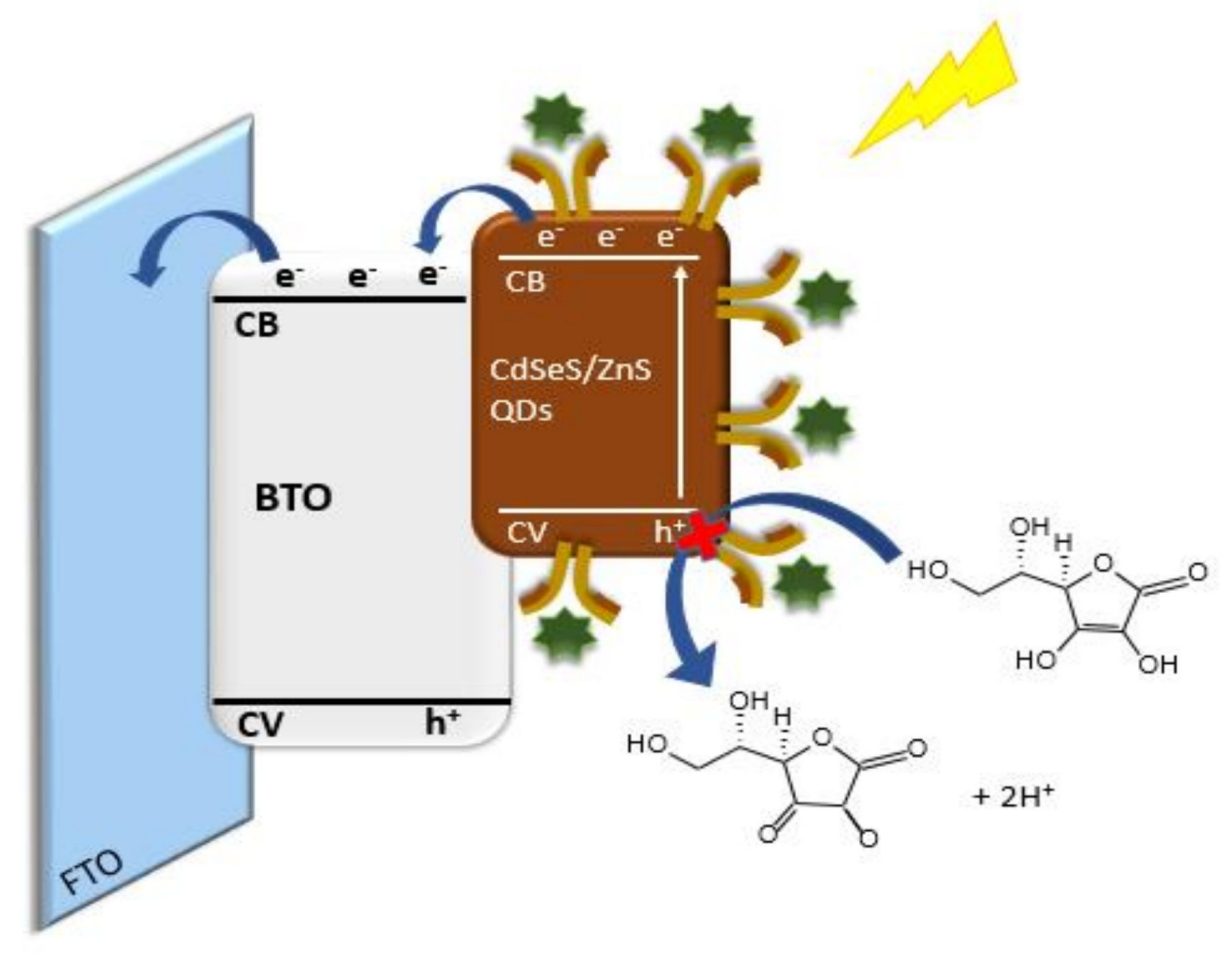
Schematic representation proposed for Mb detection using the anti-Mb/CdSeS/ZnS QDs/BTO/FTO photoelectrochemical immunosensor.

Scheme 1 shows a representation for Mb detection using the anti-Mb/CdSeS/ZnSQDs/BTO/FTO photoelectrochemical immunosensor under visible light. According to this scheme, CdSeS/ZnSQDs act as photosensitizers for BTO nanoparticles. After absorbing visible light, the QDs on the platform surface transfer electrons from their valence band (VB) to the conduction band (CB). Then, the electrons are transferred to the CB of the BTO, and collected by the FTO electrode. The VC acts as an electron donor (e^−^) to the system, suppressing the holes (h^+^) in the VB of the CdSeS/ZnSQDs, improving charge separation (e^−^/h^+^), and increasing the photocurrent. After incubating the anti-Mb modified platform with the Mb, the immunological reaction occurs simultaneously on the photoelectrochemical platform. This process blocks the capture of holes (h^+^) by the donor molecule, reducing the system’s efficiency for photocurrent generation.

### 2.4. Application of the Photoelectrochemical Immunosensor in Artificial Samples and Study of Interferents

The anti-Mb/CdSeS/ZnSQDs/BTO/FTO photoelectrochemical immunosensor was applied to artificial plasma and urine samples at different Mb concentrations to evaluate the sensor’s performance. Simulated samples of plasma (A) and urine (B) were spiked with three levels of standard concentration of Mb (10, 50, and 100 ng mL^−1^, respectively), and the immunosensor was incubated with 0.01 mL of this solution per 40 min. As shown in Table 1, the external calibration method allowed quantifications of Mb. The recovery percentages were from 95.7 to 110.7%, indicating that the anti-Mb/CdSeS/ZnSQDs/BTO/FTO platform is promising to be used to detect Mb in plasma and urine samples. In addition, the immunosensor showed a stable response in the presence of artificial plasma and urine samples after several photocurrent measurements.

**Figure 6:**
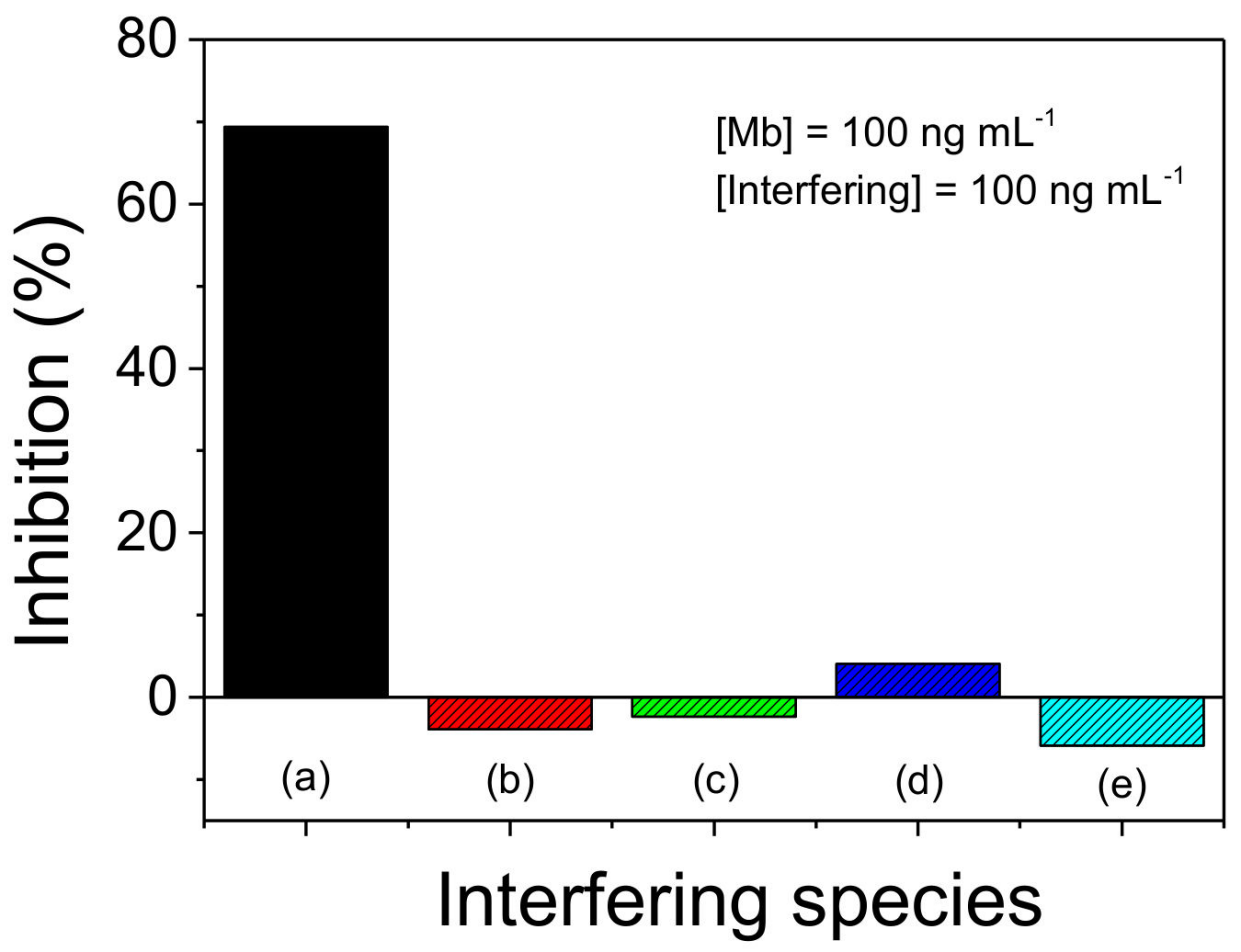
Percentage of inhibition of the anti-Mb/CdSeS/ZnSQDs/BTO/FTO immunosensor in: (a) Mb; (b) Mb + BSA; (c) Mb + HSA; (d) Mb + Troponin; (e) Mb + Cytochrome C. Measurements performed in 0.10 mol L**^−1^** phosphate buffer solution, pH 7.4, containing 0.25 mol L^−1^ of VC, under applied potential of 0 V and incubation time of 0.67 h.

Finally, out the interference study was carried to evaluate the selectivity of the photoelectrochemical immunosensor for Mb. Figure 6 shows the percentage of inhibition for the anti-Mb/CdSeS/ZnSQDs/BTO/FTO immunosensor in 100 ng mL^−1^ of Mb (a) and 100 ng mL^−1^ of the interferent species (b–e). The results show a good selectivity of the immunosensor for detecting Mb, even with the mixture of interferers with Mb. The low inhibition percentages show that the photoelectrochemical immunosensor is an excellent alternative for detecting Mb and diagnosing acute myocardial infarction.

## 3. Materials and Methods

### 3.1. Reagents

All reagents were of high purity grade. Titanium(VI) oxide, dichlorobarium, caustic potash, perhydrol 30%, hydrogen chloride, CdSeS/ZnS alloyed quantum dots (λ = 665 nm), poly(D-glucosamine), etanoic acid, glutaric dialdehyde solution 5%, albumin bovine serum 1%, sodium dihydrogen phosphate monohydrate, caustic soda, ascorbic acid or vitamin C (VC), hydrogen borate, orthophosphoric acid, sodium hydrogenphosphate, citric acid, *n*-(2-Hydroxyethyl)piperazine-*N*′-(2-ethanesulfonic acid), monoclonal myoglobin antibodies (anti-Mb) and myoglobin (Mb) were purchased from Sigma Aldrich (São Paulo, Brazil). All aqueous solutions were prepared with water distilled in OS10LXE Gehaka equipment (São Paulo, Brazil).

### 3.2. Morphological, XRD, Electrochemical, and Photoelectrochemical Measurements

Morphological characterization of the CdSeS/ZnSQDs/BTO material was performed by scanning electron microscopy (SEM) and transmission electron microscopy (TEM). SEM images were obtained using a Quanta 200 FEG (FEI-Thermo Fisher Scientific, Brno, Czech Republic) operating at an acceleration potential of 2.00 kV. TEM measurements were performed on a Tecnai G2-20 Transmission Electron Microscope (FEI-Thermo Fisher Scientific, Brno, Czech Republic) SuperTwin operating at 200 kV. The crystal structure of the materials were analysed by X-ray diffraction (XRD), using a Bruker D8 Advance diffractometer (Bruker AXS, Karushe, Germany), with a CuKα radiation tube, operating at 40 kV/40 mA, and the LynxEye linear detector. XRD measurements were performed with 2θ in the range of 10–95°, with a step size of 0.02° and counting time of 0.5 s. The program TOPAS V5 was used to perform the Rietveld refinement. 

An Autolab potentiostat model PGSTAT128N from Metrohm, equipped with a frequency response analyzer (FRA), was employed to perform the amperometric and electrochemical impedance spectroscopy measurements. These experiments were performed in the absence and presence of light. The Nova 2.1 software (Methrom Autolab, Utrecht, Netherlands) was used for photocurrent monitoring and FRA software for impedance data acquisition. A conventional cell with three electrodes: with fluorine-doped tin oxide (FTO) as the working electrode, Ag/AgCl (sat. KCl) as a reference, and a foil of gold as the counter electrode, was used to carry out these measurements. In addition, a 30 watts commercial lamp was used inside a closed homemade box as an irradiation source. Electrochemical Impedance Spectroscopy data were obtained in 0.10 mol L^−1^ of potassium chloride with 0.005 mol L^−1^ of potassium ferricyanide in a range of 10^−2^–10^5^ kHz. In addition, the proposed photoelectrochemical system is low-cost because, in this work, a visible LED lamp was purchased in local stores and the lamp was adapted in a homemade rigid box.

### 3.3. Obtention of the Barium Titanate and Modification of the Photoelectrochemical Imunossensor

Barium Titanate nanoparticles were prepared based on previous work [52]. For this, 0.80 g of TiO_2_ was dissolved in perhydrol solution (15 mL). The resulting solution was sustained in agitation using a system of sonication in ultrasound heated at 60 °C for 0.33 h. A total of 2.44 g of dichlorobarium was mixed with this solution and agitated in an ultrasound bath (for 0.17 h). Afterwards, the mineralizer caustic potash (1.00 mol L^−1^) was added. The resulting solution was stirred for a further 0.08 h, and the volume was completed at 100 mL. Then, the dispersion was placed in a Teflon bottle at 140 °C for 1 h in an atmosphere of nitrogen (25.0 bar). A Milestone Synthwave microwave reactor (Milestone, Srl, Sorisole, Italy) was employed under continuous mechanical stirring of 600 rpm, at 10 °C per min with the power of 1000 W. After this step, the reaction cell was cooled to room temperature and the BTO precipitated powder was neutralized after washing with deionized H_2_O several times; then, the material was filtered and dried in an oven at 110 °C for 24 h. Then, 0.5 g mL^−1^ of BTO was placed in the ultrasound for 0.25 h and 0.01 mL of this solution was placed onto the FTO surface. After evaporating the solvent, the modified platform was sintered on the plate at 350 °C for 0.5 h to obtain the BTO/FTO platform. After reaching room temperature, 0.01 mL of CdSeS/ZnSQDs was placed on the BTO/FTO surface to obtain the CdSeS/ZnSQDs/BTO/FTO platform. For better film fixation, 0.01 mL of poly(D-glucosamine) were placed on the film. 

First, an aqueous solution of anti-Mb of 5 × 10^−4^ g mL^−1^ was prepared, and from this solution, different solutions of the antibody were prepared (1 µg mL^−1^, 10 µg mL^−1^, 15 µg mL^−1^, 20 µg mL^−1^ and 25 µg mL^−1^). Then, a solution containing 0.015 mL of poly(D-glucosamine), and 0.02 mL of glutaric dialdehyde was prepared, and 0.01 mL were mixed with 0.01 mL of monoclonal myoglobin antibodies. Subsequently, 0.01 mL of this mixture was retired by placing it on the CdSeS/ZnSQDs/BTO/FTO platform and allowed to dry at room temperature. A total of 0.01 mL of albumin bovine serum 1% was added to the previous platform with antibodies. After drying (room temperature) for 0.25 h, the anti-Mb/CdSeS/ZnSQDs/BTO/FTO immunosensor was obtained. Then the immunosensor was washed with water to remove any unabsorbed species. Finally, for the construction of the analytical curve for the detection of Mb, 0.01 mL of Mb solutions (0.01 to 1000 ng mL^−1^) were added under the sensor’s surface, leaving them in contact with the antibody for a time of 0.67 h.

### 3.4. Preparation of Artificial Blood Plasma and Urine Samples

The system’s performance in analyzing biological fluids was evaluated by adding known concentrations of Mb to the artificial blood plasma and urine samples. The artificial plasma samples were prepared according to Liu and collaborators [53], and the artificial urine was prepared according to Laube and collaborators [54], both with minor modifications.

## 4. Conclusions

This work describes the viability of the photoelectrochemical immunosensor based on a BTO film sensitized with CdSeS/ZnS quantum dots for Mb detection, using a low-cost commercial Light Emitting Diode lamp and a homemade dark box. SEM and TEM images for CdSeS/ZnSQDs/BTO showed the presence of CdSeS/ZnSQDs clusters on the BTO surface, confirming the sensitization with these particles. Furthermore, the improved charge separation under visible Light Emitting Diode illumination provided a better photocurrent for the CdSeS/ZnSQDs/BTO/FTO platform, leading to a wide linear response range and a low detection limit. Thus, the developed anti-Mb/CdSeS/ZnSQDs/BTO/FTO photoelectrochemical immunosensor proved to be viable and successful for sensitive and accurate detection of Mb.

## Figures and Tables

**Table 1:** Results for Mb detection in artificial plasma samples (A) and artificial urine samples (B) using the immunossensor.

Sample *	Spiked (ng mL^−1^)	Found (ng mL^−1^)	Recovery (%)
A1	10	10.14 (±0.02)	101.4
A2	50	49.66 (±0.01)	99.3
A3	100	99.31 (±0.03)	99.3
B1	10	10.54 (±0.03)	105.4
B2	50	55.34 (±0.01)	110.7
B3	100	95.72 (±0.04)	95.7

* *n* = 3 (three replicates)

## Data Availability

Not applicable.

## Supplementary Materials

The following supporting information can be downloaded at: https://www.mdpi.com/article/10.3390/molecules27154778/s1, Table S1. Analytical characteristics of immunosensors for Mb and comparison of materials with the platform proposed; Figure S1: TEM images of three different regions of CdSeS/ZnSQDs/BTO material.

## Sample Availability

Samples of the compounds are not available from the authors.
