# Supplementary material for: Zero-Biased Photoelectrochemical Detection of Cardiac Biomarker Myoglobin Based on CdSeS/ZnS Quantum Dots and Barium Titanate Perovskite
Source: Molecules. 2022 Jul 26;27(15):4778. doi: 10.3390/molecules27154778 (PMC9330231; doi:10.3390/molecules27154778)
Supplement: Supplementary file 1 [file molecules-27-04778-s001.zip › molecules-1755314-supplementary.pdf]

Table S1. Analytical characteristics of immunosensors for Mb and comparison of materials with the platform proposed.

Analytical Method	Matrix	$T_{incubation}$	Working Range	LD	Ref.
SWV	AuNP-PEI	10 min	9.96 – 72.8 ng mL^{-1}	6.29 ng mL^{-1}	[3]
EIS	ab-Mb/MUA-MPA/Au	1.5 h	10 – 650 ng mL^{-1}	5.2 ng mL^{-1}	[5]
SPR	µCIP SPR	30 min	0.1 – 1.0 µg mL^{-1}	26.3 ng mL^{-1}	[11]
Chemiluminescence	Luminol–Mb–$K_4Fe(CN)_6$	-	0.1 – 100 nmol L^{-1}	0.04 nmol L^{-1}	[34]
Chronocoulometry	DNA aptamers	60 min	10 pmol L^{-1} – 100 nmol L^{-1}	10 pmol L^{-1}	[35]
ECL	RGO/ $Ru(bpy)_3{}^{2+}$/ CS	30 min	0.05 – 25 nmol L^{-1}	12 pmol L^{-1}	[36]
CV	Aptamer functionalized rGO/CNT	-	1 ng mL^{-1} – 4 µg mL^{-1}	0.34 ng mL^{-1}<br>0.034 ng mL^{-1}	[37]
CV	Ti-NT/GCE	-	0.001 – 0.1 mg mL^{-1}	1 µg mL^{-1}	[38]
DPV	DApt-CS	45 min	100 pmol L^{-1} – 40 nmol L^{-1}	27 pmol L^{-1}	[39]
FIA	MB-MWNTs	-	0.1 – 3 µmol L^{-1}	20 nmol L^{-1}	[40]
EIS	$MnO_xCoO_{y,500}$	1 h	0.01 – 2000 pg mL^{-1}	0.56 fg mL^{-1}	[41]
EIS	MIP(PSA, Myo)-SPE	60 min	1 – 20000 ng mL^{-1}	0.83 ng mL^{-1}	[42]
EIS	GQDs modified SPE	10 min	0.01 – 100 ng mL^{-1}	0.01 ng mL^{-1}	[43]
SWV	SPE/CB/DDAB	15 min	5 – 500 µmol L^{-1}	-	[44]
DPV	Apt/AuNPs/BNNSs/FTO	-	0.1 – 100 µg mL^{-1}	34.6 ng mL^{-1}	[45]
PEC [a]	g-C_3N_4-MoS_2@CdS:Mn nanocomposite	1 h	1.0 pg mL^{-1} – 50 ng mL^{-1}	0.42 pg mL^{-1}	[46]
Fluorescence [b]	CdTe-MPA-QDs	30 min	0.304-571 pg mL^{-1}	0.045 pg mL^{-1}	[47]
CV	SnO_2-QDs@3DGR	-	5.0 - 94.0 mmol L^{-1}	0.35 mmol L^{-1}	[48]
Fluorescence	WS_2 QDs	60 min	0.01–120 µg mL^{-1}	7.6 ng mL^{-1}	[49]
Photoluminescence [c]	CD-CdTe	-	0 – 2.0 µmol L^{-1}	-	[50]
CV/EIS	Mn doped TiO_2 nanoparticles	-	3–15 nmol L^{-1}	0.22 ng mL^{-1}	[51]
PEC [d] (amperometry)	CdSeS/ZnSQDs/$BaTiO_3$	40 min or 0.67 h	10 pg mL^{-1} – 1 µg mL^{-1}<br>0.01 ng mL^{-1} – 1000 ng mL^{-1}<br>$5.62x10^{-4}$ nmol L^{-1} – 0.0562 µmol L^{-1}	10 pg mL^{-1}<br>0.01 ng mL^{-1}<br>$5.62x10^{-4}$ nmol L^{-1}	This work

CV: Cyclic Voltammetry; DPV: Differential Pulse Voltammetry; EIS: Electrochemical Impedance Spectroscopy; SWV: Square Wave Voltammetry; SPR: Surface Plasmon Resonance; PEC: Photoelectrochemical. [a,c]The authors use a 500 W Xenon lamp; [b]The authors use a 150 W Xenon lamp; [d]The authors use a 30 W visible LED lamp.

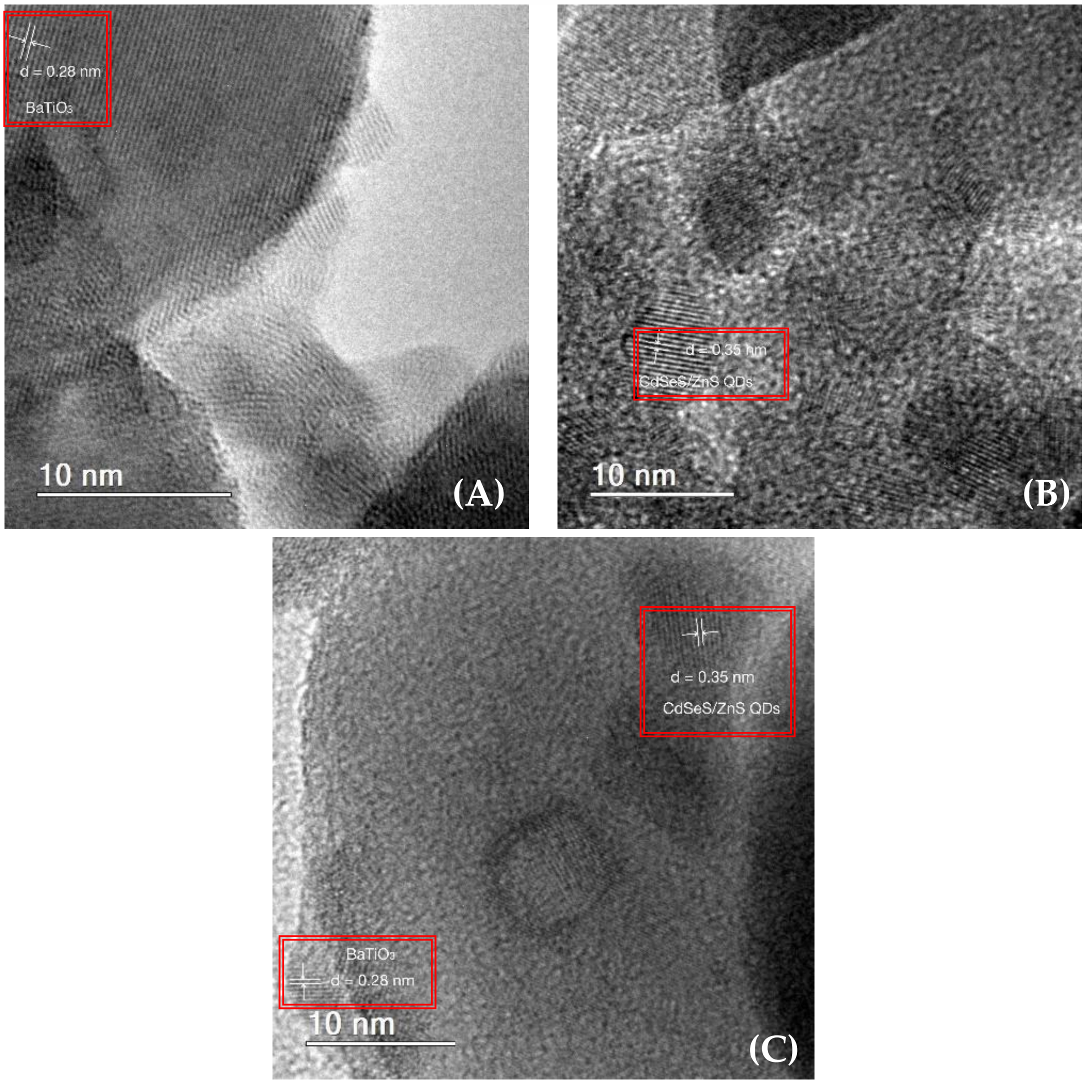

Figure S1: TEM images of three different regions of CdSeS/ZnSQDs/BTO material.
